# Capabilities of the 3D-MLSI software tool in superconducting neuron design

**DOI:** 10.3762/bjnano.17.8

**Published:** 2026-01-13

**Authors:** Irina E Tarasova, Nikita S Shuravin, Liubov N Karelina, Fedor A Razorenov, Evgeny N Zhardetsky, Aleksandr S Ionin, Mikhail M Khapaev, Vitaly V Bol’ginov

**Affiliations:** 1 Osipyan Institute of Solid State Physics RAS, 2 Academician Osipyan str., Chernogolovka, Moscow Region, 142432, Russia; 2 Moscow Institute of Physics and Technology, 9 Institutskiy per., Dolgoprudny, Moscow Region, 141701, Russia; 3 Russian Quantum Center, 30 Bolshoy Boulevard, bld. 1, Moscow, 121205, Russia; 4 HSE University, 20 Myasnitskaya str., Moscow, 101000, Russia; 5 Faculty of Computational Mathematics and Cybernetics, Moscow State University, 1-52 Leninskiye gory, Moscow, 119991, Russia

**Keywords:** adiabatic superconductor cells, inductance extraction, Josephson interferometers, multilayer niobium technology, superconductivity

## Abstract

3D-MLSI is a software tool made for inductance extraction of superconducting multilayer structures. Despite long history, its capabilities had not been explored sufficiently deep for Josephson circuits based on niobium technology. Here, we present a thorough study and verification of this program in relation to adiabatic neurons, which are extremely sensitive to variations of inductive parameters. Good agreement of experimental and extracted inductances confirms the high potential of the 3D-MLSI software package for the design of superconducting electronics components.

## Introduction

This article is devoted to one of the issues related to the design of adiabatic superconducting neurons, in particular, of sigma and Gauss neuron types [1]. They are, in fact, a single-junction and a two-junction interferometer, respectively, shunted by an additional inductance, which is also used to generate the output signal. The designation of such a neuron originates from the type of transfer (or activation) function (TF) that can be realized for a given neuron type. More specifically, the single-junction interferometer may possess a sigmoidal TF (useful for implementation of a superconducting perceptron [2]), while the two-junction interferometer may realize a Gaussian TF suitable for implementation of radial basis function (RBF) neural networks [3]. However, the desired shape of the TF is realized only for specific values of inductive parameters. This makes inductance estimation highly important for superconducting neuron design.

In fact, inductance is a crucial parameter for almost all types of superconducting electronics (SCE) circuits. Indeed, taking advantage of the high performance of SCE devices implies their operation in the gigahertz frequency range, in which incorrect circuit operation may be caused by small fluctuations in inductance. Digital and quantum SCE circuits are based on Josephson interferometers, the energy potential of which strongly depends on the inductance of the loop. For that reason, the extraction of inductances of superconducting structures has been attracting a lot of attention for many decades. Simple estimates can be made for a long line over continuous ground plane [4–5] and other primitive geometries [6]. A variety of two-dimensional (2D) programs for inductance extraction were proposed in the period of 1990–2000 (see, for example, [7–8]), which allow one to estimate self- and mutual inductances per unit length of a system of infinitely long superconducting strip lines. However, inductors of most practical devices have more complicated shapes. So, three-dimensional (3D) numerical methods are required to extract inductances needed for the design of dense and large-scale superconducting circuits.

Currently, a number of software tools have been developed that are able to simulate 3D superconducting circuits [9–13]. One of the most popular tools [14] is InductEx [9], which is based on the FastHenry engine [10] originally developed for conventional CMOS circuits [15]. In 2001, the 3D-MLSI software tool was presented [11], which is capable of extracting a three-dimensional magnetic field distribution and a planar current distribution by solving a system of integro-differential equations on a 2D grid. Recently, VoxHenry [12] and SuperVoxHenry [13] simulators were developed, which use voxel-based discretization as well as advanced numerical methods to reduce memory overhead and speed up inductance extraction. The high-frequency structure simulator by Ansys (HFSS) [16] and the Sonnet EM software [17] allow one to extract the frequency dependence of a device’s impedance. Several other methods can be mentioned that are not widely used as a tool (see, for example, [18–21]) due to the limitations on geometries and materials.

The use of a given program as a tool within computer-aided design systems (see [14,22] for reviews) requires a comparison with experimental data for validation. For InductEx, an accuracy of about 2% relative to the experimental data was reached for certain types of structures suitable for superconducting rapid single flux quantum (RSFQ) circuits [23–24]. In [13], a very good agreement was demonstrated for the newly proposed SuperVoxHenry simulator. In this article, we study the potential of the 3D-MLSI software tool [11,25], which is also a powerful inductance extractor for complex multilayer structures [22]. It seems that abilities of 3D-MLSI software package have never been checked up to now despite its well-known advantages [14,22]. Here, we present its experimental verification using two types of structures designed on the basis of low-*T*_c_ Nb–Al multilayer technology similar to that used for RSFQ-circuit fabrication. First, we compare results of experiment and simulation for simple C-shaped two-junction SQUIDs placed over a thick superconducting screen. Then more complicated objects, namely, sigma and Gauss neurons [1,26–27], are studied in experiment and simulation. Good agreement between measured and extracted values of inductances followed by the TF analysis confirms the high potential of the 3D-MLSI software package for the design of SCE devices.

## Results

### C-shaped SQUIDs

**Figure 1:**
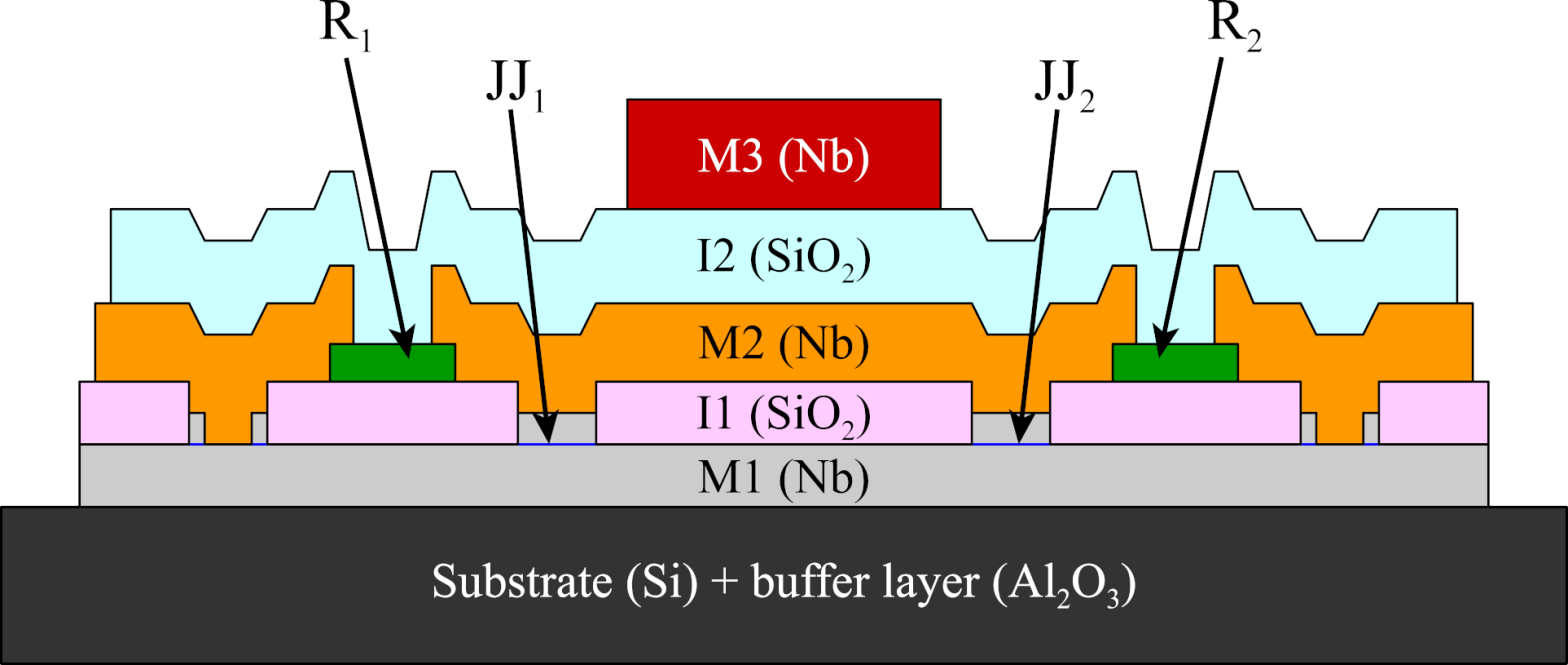
Cross-sectional diagram of a two-junction SQUID containing three superconducting layers (M1, M2, and M3) separated by insulating layers (I1 and I2). Josephson junctions are labeled as JJ_1_*_,_*_2_, and their shunt resistors are R_1_*_,_*_2_. Layer heights are to scale.

For in-depth testing regarding the capability of 3D-MLSI for inductance extraction, several series of simple C-shaped two-junction SQUIDs were fabricated and studied. The fabricated samples contained three superconducting niobium layers separated by insulating layers (see Figure 1). The first superconducting layer (M1) served both as a superconducting ground plane and the bottom electrode of the Josephson junctions. The second (M2) and third (M3) layers formed interferometers loops, control lines, and wiring. Overlap areas between layers M2 and M3 provided inductive coupling between elements. The thicknesses of the superconducting layers were 200, 250, and 350 nm for M1, M2, and M3, respectively. The SiO_2_ insulating layers had thicknesses of 200 nm (I1) and 300 nm (I2). Deposition of metallic layers was performed via magnetron sputtering in argon atmosphere, while insulating layers were thermally deposited (see parameters in [28]). The Josephson junctions (JJs) had a circular shape with 4 μm diameter (see [28] for details) and represented Nb–Al–AlO*_x_*–Nb tunnel junctions at about 100 A/cm^2^ critical current density. JJs incorporated into SQUID loops were shunted with 1 Ω molybdenum resistors to suppress capacitive hysteresis in *I*–*V* curves. Overall, this fabrication process is a type of one previously used for RSFQ logic circuits [29].

**Figure 2:**
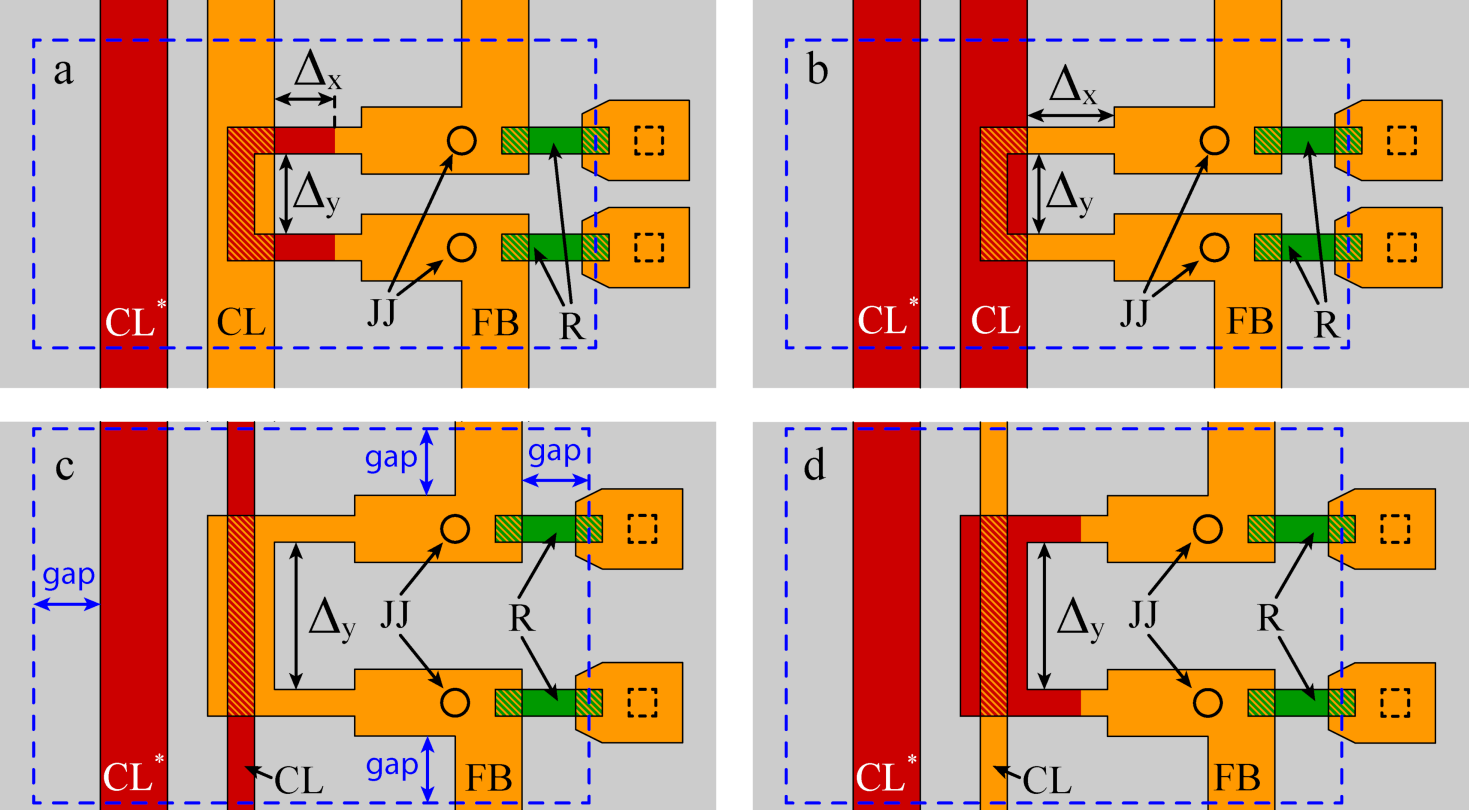
Schematics of test C-shaped SQUIDs. Gray color indicates the superconducting ground plane (layer M1), orange corresponds to the middle superconducting layer M2, and red represents the top superconducting layer M3. Inductive coupling zones (regions where M2 and M3 overlay) are shown with red hatching. Circles denote Josephson junctions. Green marks indicate shunt resistors of Josephson junctions, with hatching showing the connection of resistors to layer M2. Dashed squares indicate superconducting connections to the ground plane. Segments of variable length Δ*_x,y_* are also shown, as well as control lines CL and FB. CL* denotes an additional control line which was not used in the present experiment. Blue dashed lines demonstrate the screen size in the simulations (see next section for details). The real boundaries of the ground plane are located at a large distances from the panel edges.

In Figure 2, four types of C-shaped SQUIDs are shown schematically. Each type corresponds to one of the methods of implementation and coupling of inductive elements that can be used in neurons’ designs. In particular, inductive elements can be formed either in layer M2 (see Figure 2b,c), or in layer M3 (either fully or partially, as shown in Figure 2a,d); the control line can be wider or narrower than the SQUID loop in the coupling zone (realized by overlapping strip lines fabricated in layers M2 and M3). The designed samples included segments of variable length Δ*_x,y_* (also indicated in Figure 2), which enabled more detailed comparison with the simulation results (see below). For each design type, two series were studied, manufactured in different fabrication runs. This further expanded the possibilities for testing the 3D-MLSI software and provided insight into the reproducibility of fabrication parameters.

**Figure 3:**
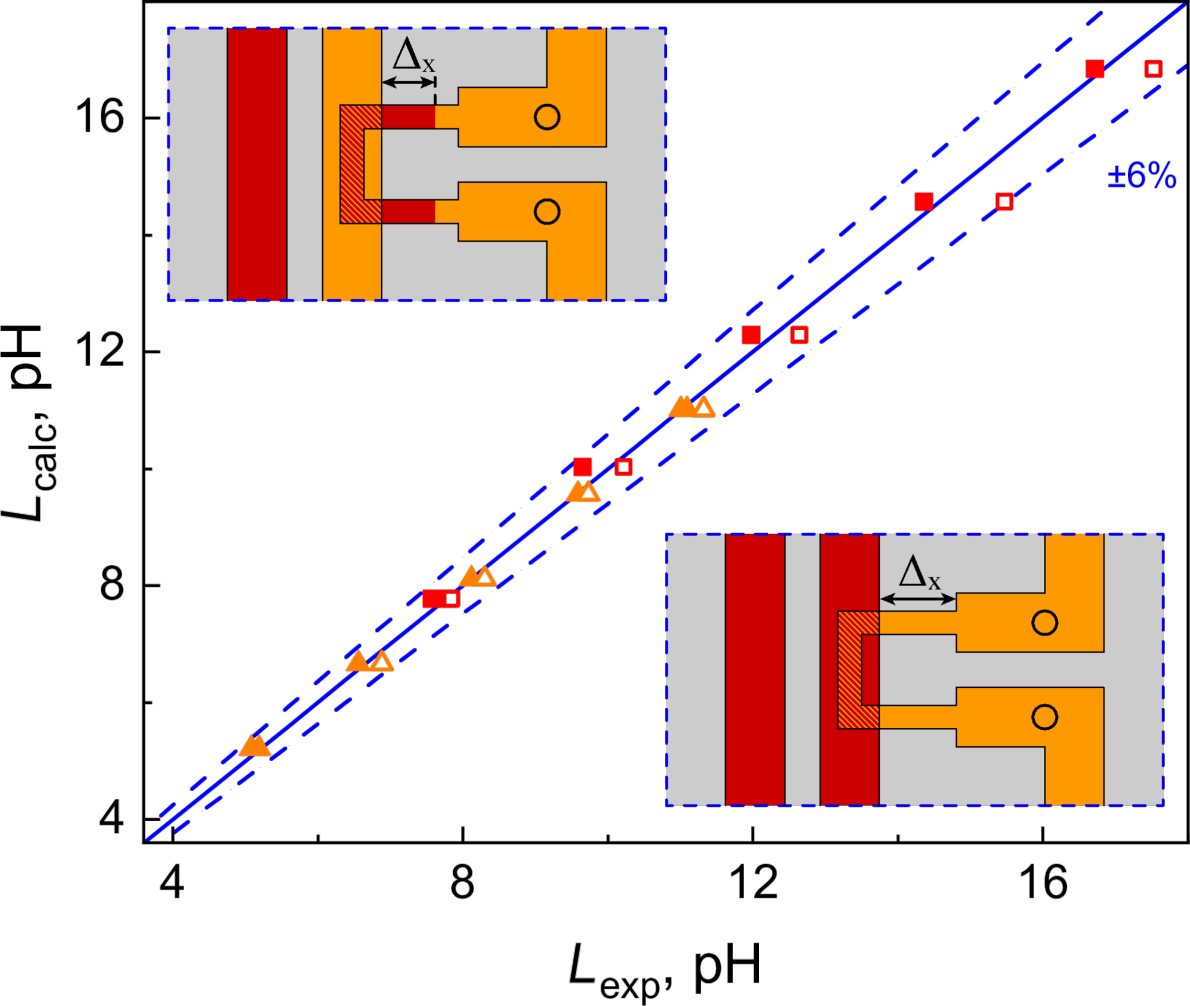
Comparison of calculated *L*_calc_ and experimental *L*_exp_ self-inductances for two-junction SQUIDs shown in Figure 2a (red squares) and Figure 2b (orange triangles). Filled and empty symbols correspond to samples obtained in the first and the second fabrication run, respectively. Identical symbols correspond to samples that differ only in Δ*_x_*. The blue solid lines represent *L*_calc_ = *L*_exp_, while dashed lines represent ±6% divergence angle.

All experiments were performed at *T* = 4.2 K with the use of a ^4^He cryostat. The main type of experiments is related to measurements of inductances of the two-junction SQUIDs. Experimental details can be found in Appendix A. Figure 3 shows a comparison of simulated and experimental self-inductance values for the simplest case, where the variable-length segment is aligned across the control line (denoted by Δ*_x_* in Figure 2a,b). In this case (see also insets in Figure 3), the variable segment has the simplest (two-layer) cross section. The simulation was performed with mesh steps of 0.125…1 μm, using a reduced-size superconducting screen truncated at a distance of 10 μm from the structure edges (see next section for details). For clarity, the data is presented as points on the (*x*,*y*)-plane, where *x* corresponds to experimental values and *y* to simulated ones. This format provides better visualization for comparing results obtained for different sample designs. Clearly, for perfect agreement, all points should lie on the line *y* = *x* (solid line in Figure 3). This cannot be the case in experiment; but, in fact, all points in Figure 3 lie within the 6% divergence angle (indicated by dashed lines) between simulated and experimental values. Such agreement should be considered good as a parameter spread of around 5% is typical even for leading RSFQ device manufacturers [30].

**Figure 4:**
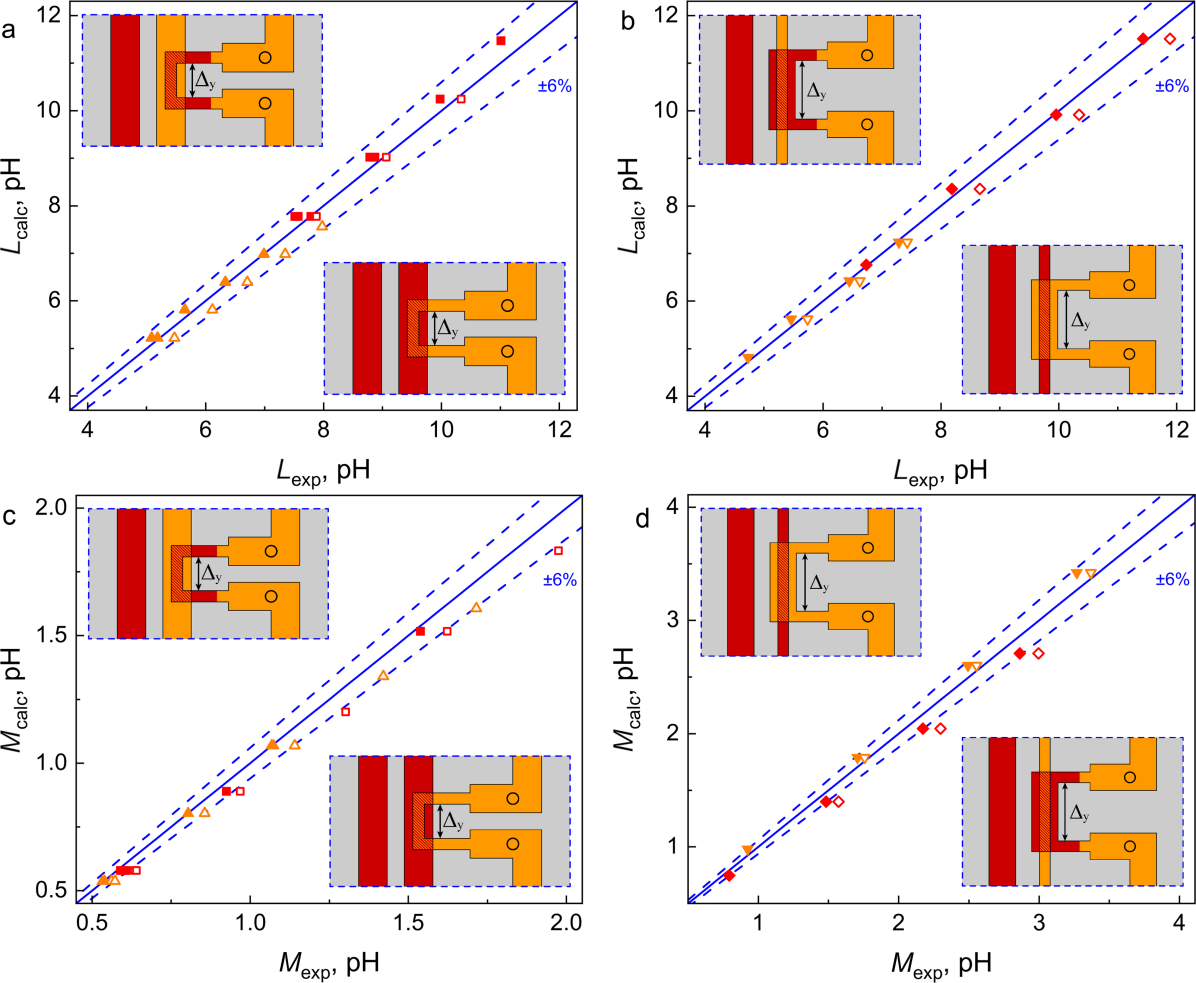
Comparison of calculated *L*_calc_ vs experimental *L*_exp_ self-inductances (a, b) and calculated *M*_calc_ vs experimental *M*_exp_ mutual inductances (c, d) for two-junction SQUIDs presented in Figure 2 and here in the insets. Identical symbols correspond to samples that differ only in Δ*_y_*. Orange triangles in panels (a) and (c) correspond to Figure 2b, while red squares in these panels correspond to Figure 2a. Orange inverted triangles and red diamonds on panels (b) and (d) correspond to Figure 2c and Figure 2d, respectively. Filled and empty symbols correspond to samples obtained in the first and second fabrication runs, respectively. Blue solid lines represent *L*_calc_ = *L*_exp_ (a, b) and *M*_calc_ = *M*_exp_ (c, d), while dashed lines represent a ±6% divergence angle.

Figure 4 shows the comparison of experimental and simulated values of self- and mutual inductances for C-shaped SQUIDs with different lengths of the coupling region. In fact, it is the design of this region that demonstrates the most significant difference among the C-shaped SQUIDs shown in Figure 2. Figure 4a,c presents data for designs where the SQUID loop has a smaller width than the control line in the overlap area (see Figure 2a,b). For brevity, one can refer to this design type as a “narrow loop”. Figure 4b,d corresponds to the “wide loop” design, where the loop is wider than the control line in the overlapping region (see Figure 2c,d). Regarding the self-inductance values (Figure 4a,b), all points fall well within the 6% divergence angle. Regarding the mutual inductances (see Figure 4c,d), the accuracy is slightly worse. Several groups of points lie on the angle boundary, and one of them falls well outside. However, even for this case, the divergence is not too large; the relative error is as small as 10% of the experimental values.

The reasons for discrepancies require additional analysis, including those related to the sample fabrication process. Note that symbols in Figure 3 and Figure 4 differ in color, shape, and filling. The color denotes the layer in which the SQUID loop is made (M2 or M3), the shape indicates the relative width of the SQUID loop and the control line, and the filling represent two series of identical samples produced in different fabrication runs. One can see that filled and empty symbols do not coincide with each other. The difference is small and does not exceed 6% of the inductances values, but it is clearly visible in the experiments. Obviously, this is due to inevitable deviations from the goal parameters during sample fabrication and, therefore, provides a criterion for good agreement between experiment and simulation. A noticeable deviation of one group of symbols from the target line (see, for example, triangles in Figure 4a) may indicate an inaccuracy in layer thickness or superconducting line width. Incomplete correspondence between the modeling and the experiment is also possible. This may be the case for red diamonds in Figure 4d since a noticeable deviation is observed for both filled and empty symbols. Fortunately, this type of coupling area design was not used in layouts of neurons studied below.

### Superconducting neurons

Next, let us consider the applicability of the 3D-MLSI program for the design of adiabatic superconducting neurons. The challenges are the more complicated shapes of inductive elements and the greater importance of inductance values for realizing the desired TF. We mainly focus on the design of a superconducting sigma neuron since its theoretical models are more developed and allow for approximation of the TF of the experimental device (see Section “Discussion”). The purpose of the sigma neuron is a sigmoidal transformation of the input signal, and the desired shape of the TF is achieved at certain values of the inductances of its loop parts (arms). The schematic of the sigma neuron was first presented in [31] (see also Appendix B). It represents a single-junction interferometer (a superconducting quantron according to [32]), the loop of which is split by an additional (output) inductance. In the simplest form, the design criterion can be stated as follows: the output inductance should divide the quantron loop into two parts with equal inductance, taking into account the effective inductance of the Josephson junction. The experimental device (see description in [26] and Appendix A) must be supplemented with elements that supply and read out the input and output magnetic fluxes, respectively. Possible interactions between parts of the experimental device complicate the analysis of its TF; however, the necessary design criteria can still be expressed through the components of a 5 × 5 inductance matrix [33].

The Gauss neuron represents a two-junction interferometer that is also shunted symmetrically to generate the output signal. This type of circuit was considered for the first time in [34–37] and named the quantum flux parametron (QFP). In [38], it was demonstrated that the energy consumption of digital SCE circuits based on QFPs can be reduced down to the fundamental limit (*kT*ln(2) per switching event). In [31], a QFP was proposed as a basic cell for RBF neural networks. Due to the more complicated form of the TF equation for the Gauss neuron, an analytical constraint for its inductances has not yet been obtained, and the optimal parameter values are selected numerically [1,31,39–42]. A generalized theory of the Gauss neuron, accounting for the interaction of all five elements of the experimental device, is currently under development by our group.

The necessity of accounting for interaction between neuron elements was revealed during the first experimental measurements of TFs presented in [26–27]. The samples were fabricated as multilayer structures above a thick superconducting screen. Experimental curves generally agreed with theoretical expectations, but included an additional linear component. One of the reasons is that the input (control line) and the readout (two-junction SQUID) elements can exchange magnetic flux via circulating currents in the superconducting ground plane [33]. It was shown in [43] that such interaction effectively results in asymmetry of the neuron’s receiving parts with respect to receiving the input signal. The corresponding component of the inductance matrix is quite small; however, it significantly affects the shape of the TF. Therefore, the designs proposed in [26–27] require further refinement.

**Figure 5:**
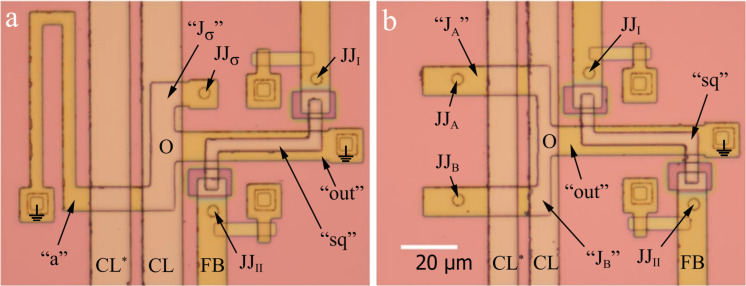
Microphotographs of investigated (a) sigma and (b) Gauss neurons. Figures show neuron's Josephson junctions JJ_A_*_,_*_B_*_,_*_σ_; the read-out SQUID consisting of the loop “sq” and Josephson junctions JJ_I,II_; control lines CL and FB; parts (arms) of neurons “J_A_*_,_*_B_*_,_*_σ_”, “a”, “out”; and the central zone “O”. Grounding symbols mark galvanic connections of the neuron to the superconducting ground plane. Boundaries of ground planes are located at large distances from the edges of the figures. CL* denotes an additional control line that was not used in the present experiment.

In this work, we study sigma and Gauss neurons whose layouts (see Figure 5) were obtained mainly by scaling down previously studied prototypes [26–27]. Some design adjustments were made also to explore the scalability of neurons layouts and to suppress screen-mediated interactions. In particular, the minimum linewidth of the strip was reduced from 10 to 4 μm thanks to a more advanced fabrication process implemented at IREE RAS [44–46]. As a result, the area occupied by the sigma neuron was reduced by a factor of 4.4 (to 10,500 μm^2^). Conversely, the area of the superconducting ground plane was increased by a factor of 5.3 (to 400,000 μm^2^). Similar modifications were applied to the layout of the Gauss neuron sample described in [27]. Its size was reduced by a factor of 4.7 (to 8,050 μm^2^), and its ground plane area was increased by a factor of 6.3 (to 400,000 μm^2^). The neurons were placed in the center of the screen with their output arm, which has the same shape for sigma and Gauss neurons, aligned along the symmetry axis of the screen. Suppression of screen-mediated interaction was expected due to the inverse proportionality of the coupling to the transverse (relative to the control line) size of the screen [26].

Experimental TF and their analysis will be discussed in the Section Discussion. Here, we consider extraction of inductances necessary for TF analysis. To do this, a series of specific samples were made and studied, which are two-junction SQUIDS based on partial loops of the sigma neuron. Note that the loop of either neuron in Figure 5 consists of three arms ("J_σ_", *L*_"a"_, *L*_"out"_ in Figure 5a and “J_A_”, “J_B_”, *L*_"out"_ in Figure 5b) and has three connection points to the ground plane. To transform the neuron into a two-junction SQUID, one of the connections must be opened, while the other two should be closed via Josephson junctions. This can be done in three ways, while the fourth interferometer type is a readout SQUID coupled to a neuron in which all three arms are opened. Experimental and numerical studies of partial loops inductances provide values necessary for further substitution into theoretical formulas. Details of neurons decomposition and inductance calculations are given in Appendix B.

**Figure 6:**
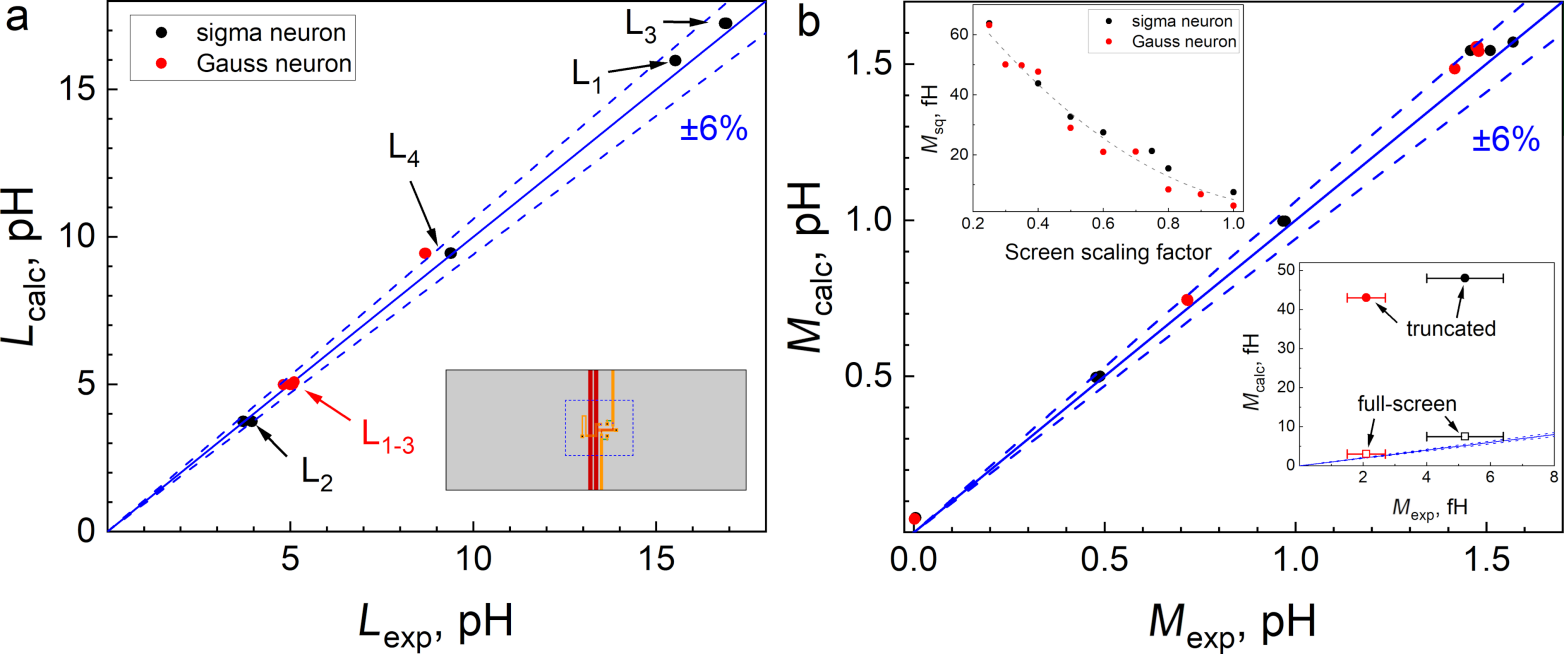
Comparison of calculated (*L*_calc_ and *M*_calc_) and experimental (*L*_exp_ and *M*_exp_) values of self- (a) and mutual (b) inductances of partial loops of sigma (black dots) and Gauss (red dots) neurons (see definitions in Appendix B). Solid blue lines represent *L*_calc_ = *L*_exp_ and *M*_calc_ = *M*_exp_, while dashed lines represent the ±6% divergence angle. The inset in panel (a) shows a sigma neuron placed on the full-size ground plane with blue dashed lines designating the boundary of the truncated ground plane used in simulation. The upper inset in panel (b) shows the dependence of *M*_sq_ on the truncated screen size (see details in the text). The lower inset shows the section of the diagram located near the origin. Filled and empty symbols corresponds to simulation on the truncated and full screen, respectively.

The fabrication process was the same as described above. All inductive arms of the neurons were formed in the M2 layer, while the control line and the loop of the readout SQUID lied mostly in the M3 layer. Thus, when studying the interaction of neuron arms with the control line, the inductive coupling was implemented like in the “narrow M2” test SQUIDs (Figure 2b). When measuring the mutual inductances of neuron arms to the readout SQUID, an inductive coupling type “wide M2 loop” was realized (Figure 2c). Experimental investigations were performed as described in Appendix A. Numerical simulations were carried out assuming a truncated superconducting screen with a gap between the structure and the screen edge of 50 μm. Thus, the screen size (225…170 μm × 243…177 μm) was larger compared to test C-shaped SQUIDs, which led to an increase in the main mesh step to *ah* = 2 μm and the edge step to *ahb* = 0.25 μm in order to meet the limitations on the amount of allocated RAM and execution time (see Section “Extraction Details”). Results are presented in Figure 6 as a dependence of calculated vs measured values, similar to Figure 3 and Figure 4. One can see that almost all experimental points fall within the 6% divergence angle for self- and mutual inductances for partial loops of both types of neurons.

A couple of points in Figure 6b can be found that are noticeably out of the 6% divergence angle (see “truncated” points in the lower inset on Figure 6b). However, there are several objective reasons for this. First, these experimental points are located in the vicinity of the origin, where the area determined by ±6% divergence angle is very small in absolute units. Second, these points correspond to the smallest inductances *M*_sq_ that describe the parasitic screen-mediated interaction between the control line and readout SQUID of experimental neurons. These points lie above the 6% divergence angle, indicating that actual *M*_sq_ values are substantially smaller than the calculated ones. This is explained by the high sensitivity of *M*_sq_ to the size of superconducting ground plane. In fact, the screen-mediated interaction is determined by the ring currents circulating in the screen to close the return current caused by the magnetic field of the control line. Obviously, the forced truncation of the ground plane in simulations (see lower inset in Figure 6a) can greatly affect the distribution of the ring currents. This is demonstrated in the upper inset on Figure 6b, which shows the dependence of the calculated parasitic inductance on the screen scaling factor (the ratio of the size in simulation to the real size, same for both lateral dimensions) at *ah* = 4 μm and *ahb* = 0.5 μm. As the scale factor increases, the result tends to values of about 3–7 fH, which agrees with the measured values by an order of magnitude. That is why “full-screen” points in the lower inset in Figure 6b lie much closer to the divergence angle.

The measured *M*_sq_ values, in turn, are 10–20 times less than estimated in [26,47]. This is probably due to the increase in the size of the superconducting screen, which was intended just to suppress screen-mediated interaction. Thus, at least one way to dump screen-mediated interaction exists, and this type of coupling is not an impassable barrier on the way to implementation of superconducting neurons. Increasing the neuron integration density in practical devices can be achieved by expanding the screen to cover the entire substrate area. In this case, the actual size of each neuron will be determined by the outer boundaries of its elements (arms). Potential challenges of this approach will be addressed in our future publications.

As was proposed in [47], another possible way to dump the screen-mediated interaction is the use of an additional control line CL* (see Figure 2 and Figure 5), which is located near the main control line CL and carries the same control current in the opposite direction. This allows for localization of the circulating currents between CL and CL*, diminishing their effect on other elements. However, this method could not be tested in the present work due to the high efficiency of the previous one. Indeed, the actual values of these inductances are extremely small (about 2–5 fH) and correspond to the limit of sensitivity of our experimental technique. Therefore, the relative error of such measurements is too high and any conclusion would not be reliable enough.

## Extraction Details

The primary subject of this article is the detailed verification of 3D-MLSI software tool. The main task of this inductance extractor is an evaluation of two-terminal partial inductances [48] associated with equivalent scheme ones. The general mathematical model for all superconductor inductance calculations are Maxwell and London equations with proper excitation. Based on them, the inductance can be evaluated using the free energy functional. Therefore, the basic equations for 3D-MLSI are static London and Biot–Savart expressions for magnetic field 
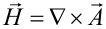
, vector potential 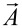, and full energy *E*. The only free parameter here is the London penetration depth [5], which was taken as 85 nm according to manufacturer data.

Details of 3D-MLSI numerical technique can be found in [49–50]. An input data file, specified in text format, must contain the geometry of the device in the plane of the substrate, the parameters of the superconducting layers (thicknesses, relative positions, and London lengths) as well as commands related to the numerical process. This internal format is different from the conventional representation of design in the form of GDSII and DXF data. However, this data can be easily converted into 3D-MLSI format using, for example, the KLayout editor [51]. The input file should also specify current paths through terminals to enable calculation of the partial inductances. The supercurrent can be transmitted between layers using internal current sources as described in [49]. 3D-MLSI contains native support for currents around holes (moats) as well as evaluation of the related inductances. Several improvements have been made compared to the previous [49] version of the program. First, OpenMP multithreading was implemented for computationally heavy procedures. Second, easy support for non-planarized processes was developed (“nonplanar” option), where the height of a wire can vary in-plane (see Figure 1). Third, input data preparation was simplified, which allows to present conductors as polylines.

The distinctive feature of 3D-MLSI is an advanced finite element method (FEM), a numerical technique based on averaging the 3D current over the thin thickness of a superconductor film [49–50]. In contrast to InductEx and SuperVoxHenry, it leads to a set of 2D integro-differential equations instead of three-dimensional ones. As a result, 3D-MLSI can work without the large matrix procession techniques implemented, for example, in the SuperVoxHenry tool (e.g., fast-Fourier tensor acceleration, Tucker decompositions, fast multipoles method, and the AGMG-based sparse preconditioner for fast convergence). Instead of that, 3D-MLSI FEM brings the solution to the direct filling of two dense matrices of large size, that is, a matrix for interactions between mesh cells and a Galerkin matrix for solution of integro-differential equations. Filling the matrices needs *O*(*N*^2^) operations, and the solution procedure needs *O*(*N*^3^) operations, where *N* is the number of mesh nodes. These two operations basically define the total time and memory needed for calculations. In practice, the *O*(*N*^2^) part can be comparable with the *O*(*N*^3^) solution time for moderate *N* values. Advanced matrix compression methods can be implemented in future versions to be used for dense multilayered schemes of large sizes.

**Figure 7:**
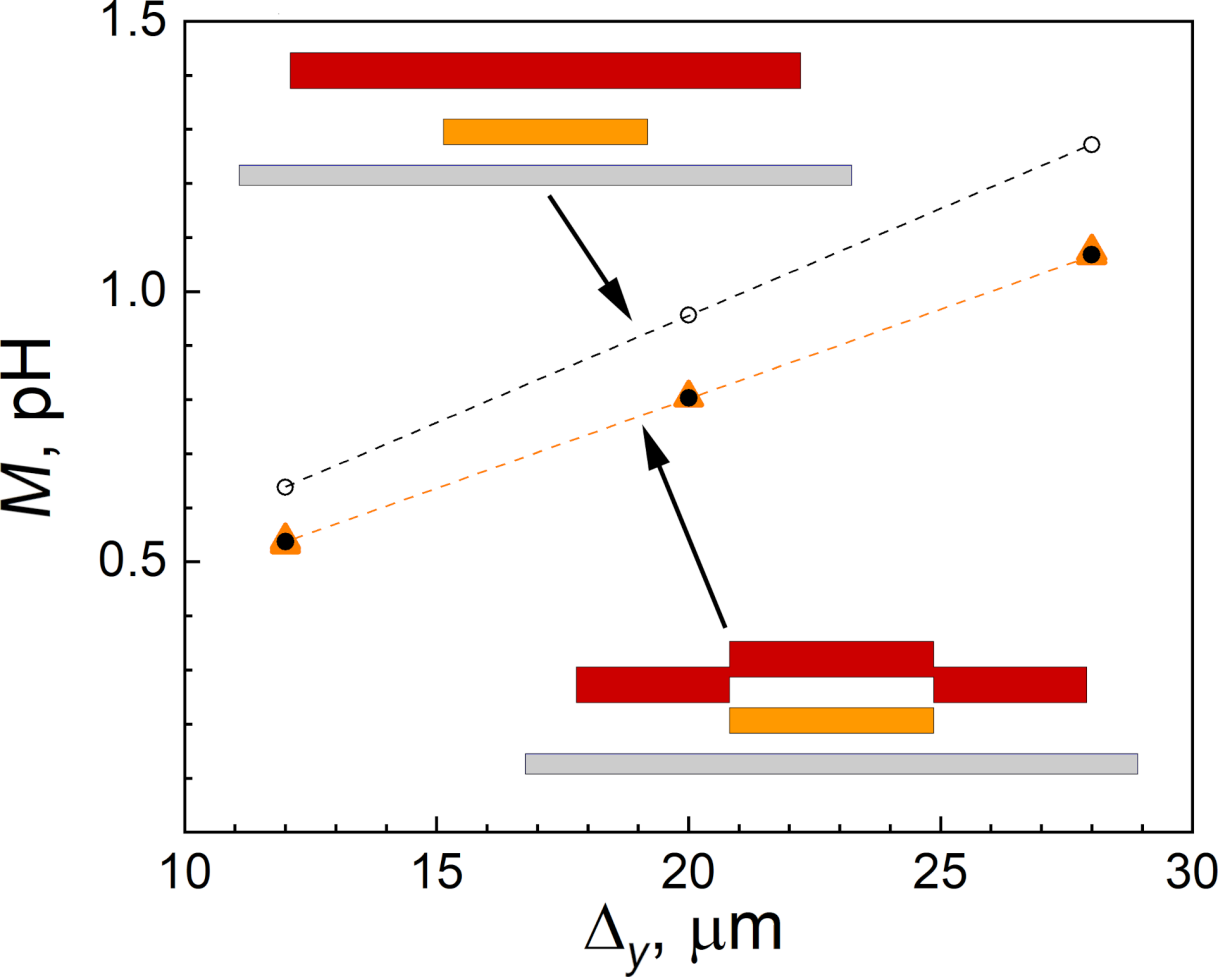
Mutual inductance dependence on the Δ*_y_* variable part length for the test SQUID sample presented in Figure 2b. Orange triangles show experimental data, filled black dots correspond to calculations with non-planar cross section (lower inset), and empty black dots were calculated with planar cross section (upper inset).

A good agreement with the experiment for all investigated structures was mainly achieved by the consideration of the in-plane coordinate dependence of the metal layer heights, which is implemented in the current version of the program as a “nonplanar” option. Figure 7 presents a comparison of experimental data with simulation results obtained with and without the “nonplanar” option enabled. The comparison was performed using the “narrow M2 loop” structure fabricated in the first run (see Figure 2b). One can see that the simulation results without the “nonplanar” option overestimate the experimental values by approximately 20%. In contrast, when the curvature of the M3 layer is taken into account, the experimental and calculated points show excellent agreement. Thus, accounting for the curvature of the metallic layers is an important condition for improving the extraction accuracy. This factor can only be neglected when the dielectric layers are planarized, as is done, for example, in the process described in [6].

**Figure 8:**
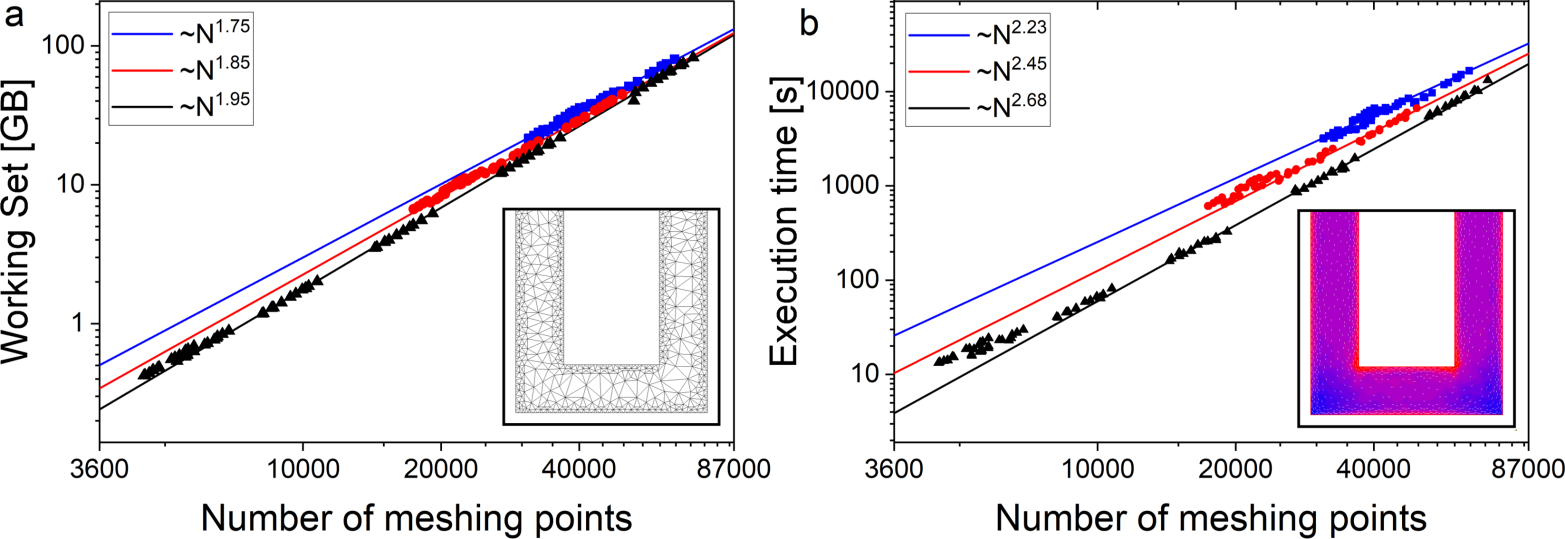
Dependence of (a) the allocated memory and (b) the execution time on the number of meshing points for a set of simulated structures (14 two-junction interferometers obtained by the decomposition of neurons as described in Appendix B). The change in the number of grid points is achieved by changing the step *ahb* at constant *ah*. Blue, red, and black dots correspond to *ah* values of *ah* = 1.5, 2.0, and 4.0 μm, respectively. Straight lines represent a power-law fit with exponent given in the legend. The inset in panel (b) shows an example of meshing produced by Triangle meshing engine. The inset in panel (a) shows the current distribution in the sigma neuron arm pivot area obtained in 3D-MLSI simulation. Red areas correspond to higher current density.

One more important feature of the program is non-uniform meshing related to the Triangle meshing engine [52]. It allows for a reduction of allocated memory and solution time, and provides better accuracy as well. Calculations are performed on a highly graded mesh of triangular cells (see inset on Figure 8a) based on two mesh step parameters. The first parameter, *ah*, defines the size of triangular cells inside the superconducting film far enough from the nearest boundary. The other parameter, *ahb*, is related to the cell size in the vicinity of the boundary. This allows for more accurate modeling of regions of strong current density changes located just in the vicinity of strip line edges (see inset on Figure 8b). The choice of the upper grid scale value *ah* is defined mainly by the minimal strip line width according to manufacturer’s design rules. So *ah* can hardly be chosen larger then 2 μm since the minimal width was 4 μm for all samples studied here.

**Figure 9:**
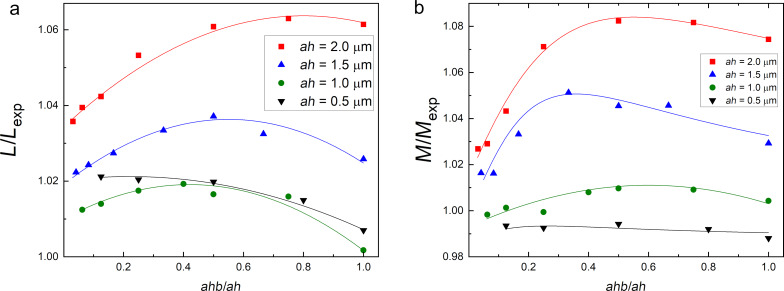
Dependence of calculated self- (a) and mutual (b) inductances of the test two-junction interferometer (see Figure 2b) on the edge discretization step *ahb* for several values of the general spatial discretization step *ah* (indicated in the Figure). Calculated values are normalized to the experimental ones.

In principle, the lower grid scale *ahb < ah* should be related to the London penetration depth λ = 85 nm for all superconducting layers. However, *ahb*, and *ah* as well, must not be too small since they define the amount of required memory via the number of mesh nodes. The working set of RAM used by the numerical core of 3D-MLSI depends on the number of meshing nodes in a power-law manner (see Figure 8a). The power of the dependence is slightly below two and depends on the grid step *ah*, which is related to the complexity of assembling the matrix *O*(*N*^2^). Thus, for the most accurate calculations performed on structures of the smallest size (C-shaped two-junction SQUIDs, Figure 2), the smallest steps *ah* = 1 μm and *ahb* = 0.125 μm (default parameters) were chosen. For certain interferometer designs, this allows for the extraction of self- and mutual inductances to be achieved with an accuracy of 1–2%, as shown in Figure 9. When modeling large SQUIDs based on partial loops of neurons, the values *ah* = 2 μm and *ahb* = 0.25 μm were mostly used. One can see that a relative accuracy within 5% can be achieved at *ahb* = *ah*/8 for *ah* ≤ 2 μm. A reasonable estimate can be obtained even for *ah* as large as 4 μm if *ahb* is substantially small. In particular, for *ahb* = 125 nm the error did not exceed 15% of the experimental value.

**Figure 10:**
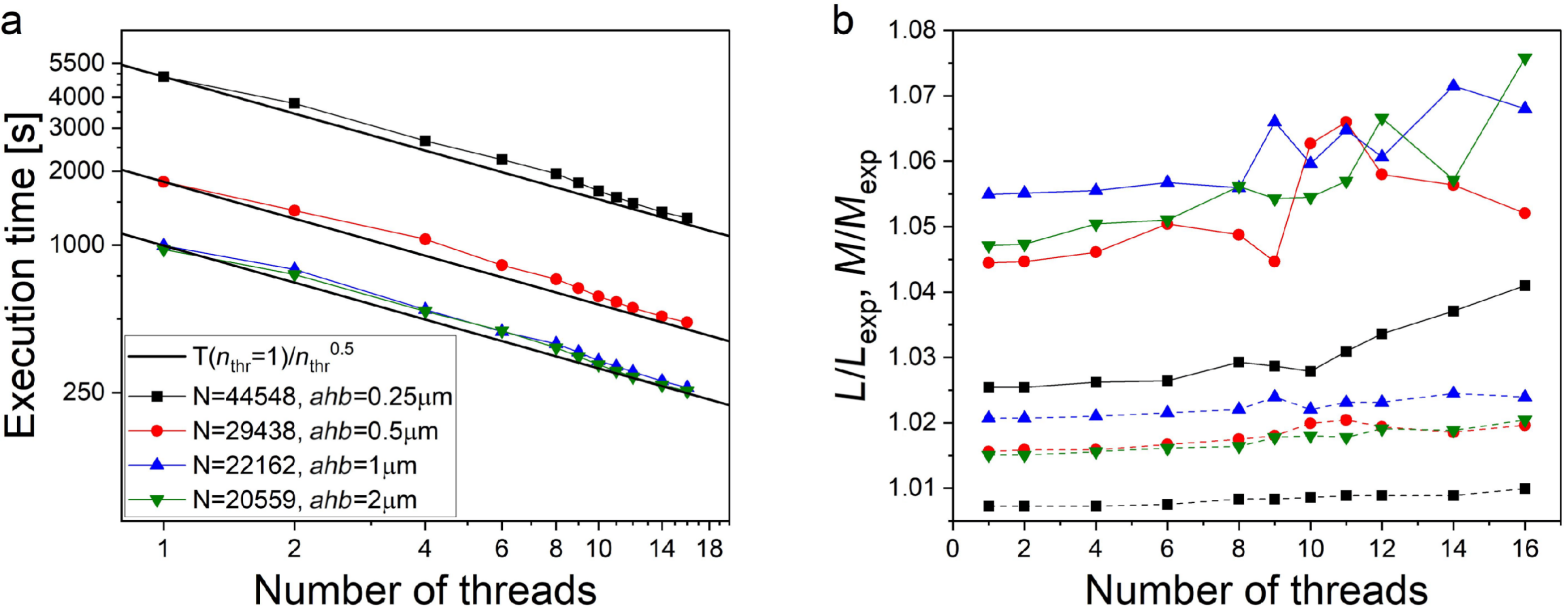
Results of OpenMP multithreading testing as applied to the simulation of the second partial loop of the sigma neuron (see Appendix B for definition). Step *ah* = 2 μm is fixed, while *ahb* varies, causing a change in the number of meshing points *N*. (a) Execution time-dependence on the number of threads at different edge discretization steps *ahb*. Solid lines represent the ratio of the program execution time in the single-threaded mode *T*(*n*_thr_ = 1) to the square root of the number of threads 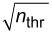. Different symbols denote simulations with various mesh steps *ahb*. (b) Dependence of estimated mutual (solid connecting lines) and self- (dotted connecting lines) inductances on the number of threads used. Simulation results are normalized to the experimental value.

An increase in the number of mesh nodes affects not only the required RAM volume but also the total computation time. Figure 8b shows that in single-threaded mode, the calculation time follows a power-law dependence on the number of mesh nodes with the power ranging from two to three, depending on the main discretizations step *ah*. The maximum number of mesh nodes reached approximately 65,000 at *ah* = 1.5 μm and *ahb* = 0.25 μm when simulating the third partial loop of the sigma neuron (see Appendix B for definition). The computation time in this case was about 7.5 h on an AMD Ryzen 9 5900X 12-core Processor with 128 GB RAM. In the current version of 3D-MLSI, the calculation time can be reduced using OpenMP multithreading. Figure 10 shows the results of calculations performed with different numbers of threads for the design of the second partial loop of the sigma neuron. To test multithreading on meshes with varying numbers of meshing points, the design was simulated with varying *ahb* at a fixed *ah* = 2 μm. Simulations were performed on an Intel Core(TM) i9-13900KF with 128 GB RAM and 24 cores (16 efficiency cores and 8 performance cores). When OpenMP multithreading is used, the computation time depends on the number of threads *n*_thr_ approximately with inverse square root law (see Figure 10a). The deviation from the expected 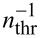 scaling indicates incomplete parallelization related seemingly to the use of the Cholesky decomposition method. With an increasing number of threads, the computed value slightly increases, within 1% for self-inductances and 2% for mutual inductances (see Figure 10b). Nevertheless, in this work, the multithreading option was not used in order to achieve maximum calculation accuracy.

**Figure 11:**
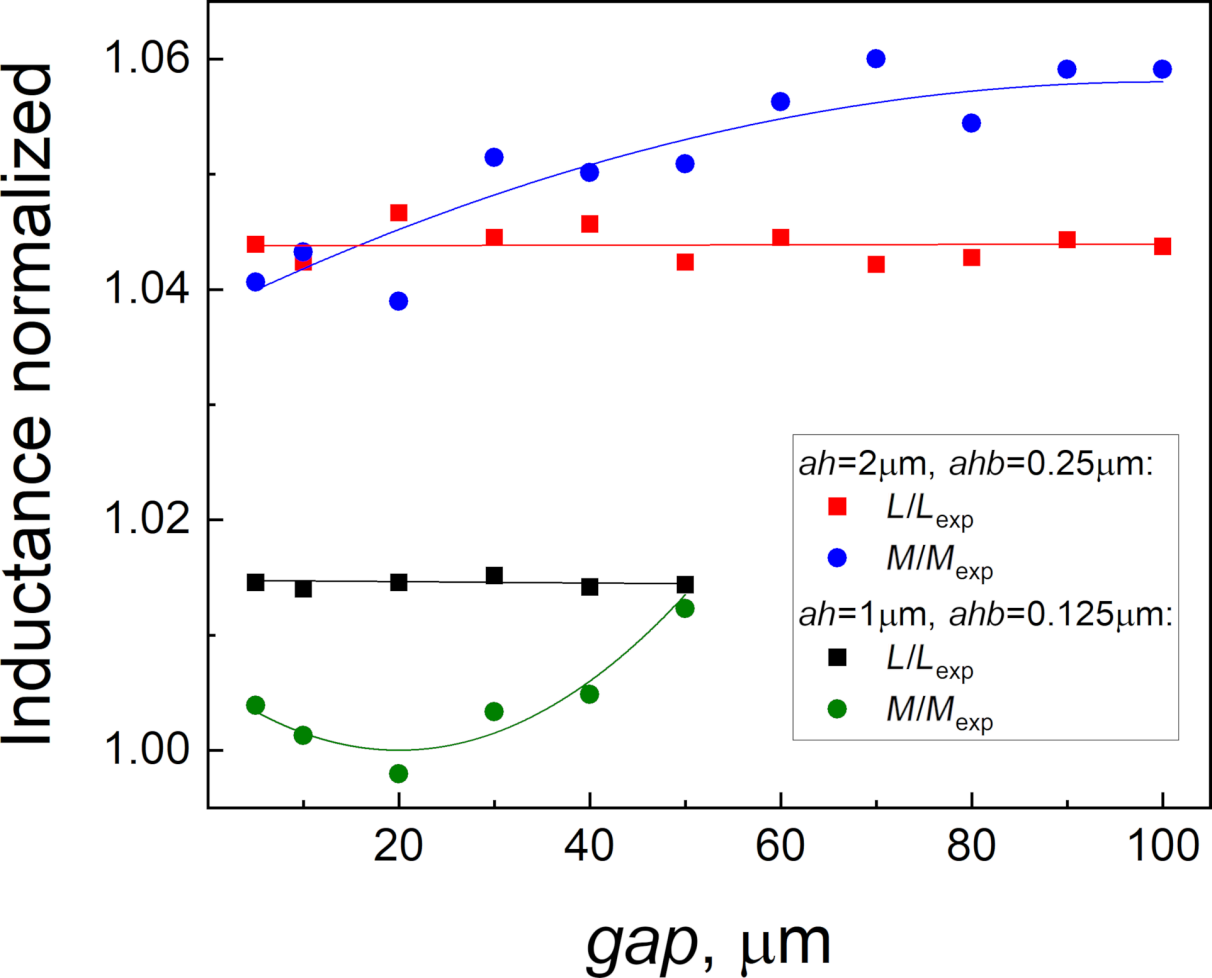
Dependence of self- and mutual inductances of the test SQUID (see Figure 2b) on the size of the superconducting screen in simulation. The horizontal axis corresponds to the distance from the outer boundaries of the structure to the truncated screen boundaries in simulation (see Figure 2c for definition).

The increase in the ground plane size, motivated by physical considerations, made it impossible to simulate the actual structures with optimal mesh steps (*ah* = 1 μm, *ahb* = 0.125 μm). Indeed, according to the data in Figure 8, the simulation of, for example, the sigma neuron would require a mesh consisting of more than 650,000 nodes, more than 53 days of computation time, and definitely more than 3.6 TB of RAM. Therefore, it was necessary to “trim” the screen for the purpose of simulation. The relevant parameter here is the gap (see Figure 2c) between the structure and the screen edge. Figure 11 shows the dependence of the simulated inductance of the test SQUID (see Figure 2b) on the gap parameter. The structure size was 64 μm × 26 μm; so the screen size varied from 74 μm × 36 μm to 264 μm × 226 μm. The obtained values of the self- and mutual inductance were normalized to the experimentally measured ones. It can be seen that for *gap* ≤ 50 μm, the deviation of the simulation results from the experimental values is only about 1–2% for *ah* = 1 μm and *ahb* = 0.125 μm. These parameters were used in Section “Results – C-shaped SQUIDs” for simulating test SQUIDs with a gap parameter of 10 μm. The maximum allowable gap (50 μm for the given *ah* and *ahb*) was determined by the available RAM (128 GB). Increasing *ah* to 2 μm and *ahb* to 0.25 μm makes it possible to increase the gap to 100 μm, though this results in a deviation of 4–6% from the experimental values. Full-screen calculations were performed only to evaluate screen-mediated coupling, which is very sensitive to the superconducting screen size. For this type of calculations, a set of parameters *ah* = 4 μm and *ahb* = 0.5 μm was used, which provides a reasonable estimate, as was stated above.

Summarizing, 3D-MLSI evaluates two-terminal partial inductances of thin multilayer structures, with the only free parameter being the London penetration depth. A fairly good performance rate is achieved due to the use of a set of 2D integro-differential equations instead of three-dimensional ones and non-uniform meshing based on the “Triangle” meshing engine. A good accuracy of simulation was reached for the mesh with the cell size varying between 0.125 and 1 μm, as was set by parameters in the input file. We have shown that accounting for the layers’ height in-plane coordinate dependence is an important condition for a good agreement with experiment. This was done using newly the added “nonplanar” option. One more helpful new feature is “nmthreads” option, which reduces calculations time using OpenMP multithreading. The limitation on the minimum mesh cell size is defined by the amount of memory ready to be allocated. To meet this limitation, a truncation of the superconducting screen can be made, which slightly affects simulated values of self- and mutual inductances, although it strongly modifies the screen-mediated coupling, as was described in Section “Results – Superconducting neurons”.

## Discussion

At present time, we continue the investigation of 3D-MLSI abilities, and new data (if any) will be published elsewhere. However, a good agreement between experiment and simulation can already be seen now. Therefore, it is interesting to consider the possibility of predicting the TF of a sigma neuron, the implementation of which is one final purpose of our studies. In [1], a simple parametric expression for the sigma neuron TF was obtained theoretically, which was further elaborated in [26] to account for the method of output signal measurement and for the interaction between input and readout elements. Taking all modifications into account, the theoretical formula takes the form:


[1] $$I_{\text{cl}}=k_{1}I_{\text{c}}\left(\varphi +k_{2}\sin\varphi -\varepsilon \right),$$

[2] $$I_{\text{fb}}=k_{3}\left(\left(k_{4}+\Delta k_{4}\right)I_{\text{cl}}-I_{\text{c}}\sin\varphi +I_{\text{c}}\zeta \right),$$

where *I*_cl_ is the control current (input signal), *I*_fb_ is the compensating (“feedback”) current (i.e., output signal), *I*_c_ is the critical current, φ is the phase difference across the Josephson junction, and the coefficients *k_i_* (as well as Δ*k*_4_) are expressed in terms of the self- and mutual inductances of the neuron arms. The “offset terms” ε and ζ depend on the initial flux in the readout element and do not affect the shape of the TF. That is why we used them as free parameters for fitting the experimental curve.

**Figure 12:**
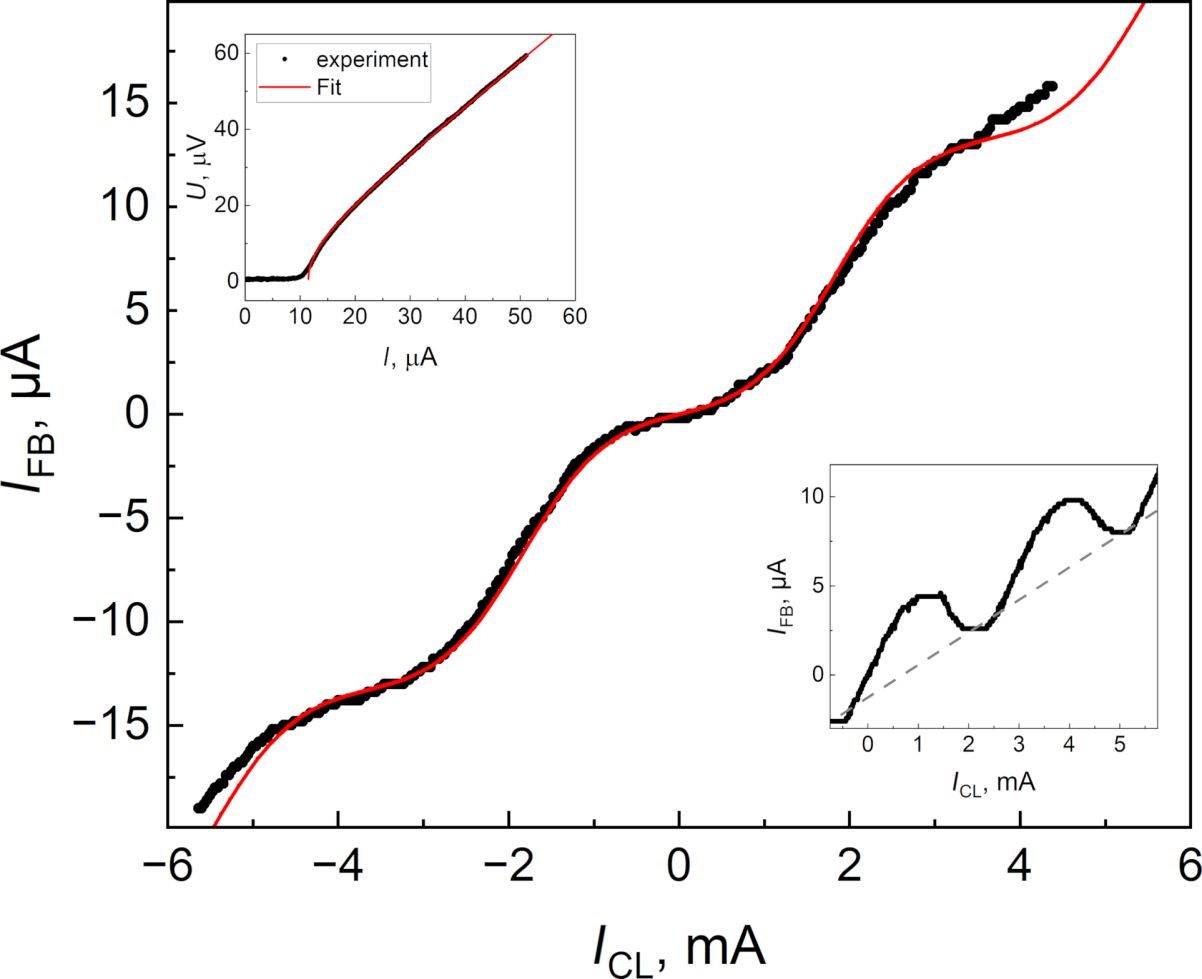
Comparison of the experimental (black dots) and the approximated (solid red curve) TFs of the sigma neuron (see Figure 5a). The upper left inset presents the measured voltage–current characteristic for a test SIS junction and RSJ fit with *I*_c_ = 12 μA critical current. In the bottom right inset, the measured TF (black dots) of the Gauss neuron (see Figure 5b) is given. The dotted line corresponds to the linear slope caused by the screen-mediated interaction between control and readout parts of the device.

The experimental TF of the sigma neuron (and the Gauss neuron as well) was measured at *T* = 4.2 K using flux compensation technique described in [26] (see also Appendix A). This curve was fitted by the dependence in Equation 1 and Equation 2 with values *k_i_* evaluated from the arms inductances. The necessary quantities were calculated using self- and mutual inductance values of the neurons’ partial loops, both measured experimentally and extracted with the 3D-MLSI program (see Figure 6 for comparison and Appendix B for details on partial loops). We had to use Δ*k*_4_ as a free parameter since it depends on the indirect coupling value *M*_sq_, which could not be reliably measured or calculated. Then, *M*_sq_ was calculated on the basis of the Δ*k*_4_ fit value with further comparison with the above results. One can see that experimental and numerical curves coincide rather well in Figure 12. The extracted value of *M*_sq_ was 10.5 fH, which is consistent with both experimental and numerical estimates obtained above. One more fitting parameter could be the critical current *I*_c_, whose value cannot be measured directly and may, in principle, vary slightly from one junction to another. Nevertheless, a good agreement between calculated and experimental results was obtained using a value of 12 μA, which agrees with the experimentally measured one on a test SIS junction (see the upper inset in Figure 12). Thus, the high potential of the 3D-MLSI software for designing artificial superconducting neurons is clearly confirmed.

The TF of the Gauss neuron was measured as well (see the lower inset in Figure 12); however, it cannot be fitted at the moment due to lack of theory accounting for interaction of all of the Gauss neuron parts. Some analysis can be made based on recently presented results [43]. Similarly to earlier experiments [27], the TF represents a bell-shaped curve based on a tilted line. The slope of the line is defined by a real or effective asymmetry of the input arms couplings 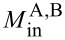 to the incoming signal. The asymmetry can be characterized by the parameter 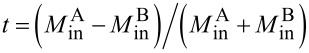 and results in an unequal supply of the input flux in proportions (1 ± *t*)Φ_in_/2 to the receiving arms J_A,B_. As a consequence, a part of the input signal *t*Φ_in_/2 is mixed to the output one Φ_out_, resulting in the undesired linear component of the TF. This type of asymmetry was noted in [47] when simulating the older sigma neuron design [26], despite identical shape of its receiving parts. Here, both experiment and simulation have shown the absence of coupling asymmetry (
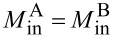
, *t* = 0 within several percent uncertainty), seemingly, due to the increase in the screen size. Nevertheless, screen-mediated interaction between the input (“CL”) and readout (“sq”) elements can give the same effect, with an effective asymmetry parameter [43]




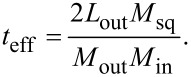




Based on experimental measurements (*L*_out_ ≈ 2.40 pH, *M*_sq_ ≈ 2 fH, *M*_out_ ≈ 1.47 pH, *M*_in_ ≈ 1.43 pH), we estimate *t*_eff_ ≈ 0.005. Using calculated values (*L*_out_ ≈ 2.45 pH, *M*_sq_ ≈ 3 fH, *M*_out_ ≈ 1.55 pH, *M*_in_ ≈ 1.48 pH), the estimate gives *t*_eff_ ≈ 0.006. Despite the small value of *t*, the effective asymmetry significantly distorts the TF, likely because of the really small amplitude of the output signal. Therefore, the presented design of the Gauss neuron require further improvement. By now, an increase in sensitivity to the input signal and in efficiency of the output flux transfer, as well as a suppression of parasitic coupling, seems to be the most promising strategy. The potential of 3D-MLSI software demonstrated in this work will be useful in this process.

## Conclusion

Capabilities of the 3D-MLSI software tool were thoroughly tested for several designs of practical multilayer superconducting structures. Advantages of the numerical methods, non-uniform triangular meshing, non-planarized superconducting layers support, and OpenMP multithreading were demonstrated aimed at the enhancement of accuracy and performance. An agreement as good as ±6% of the experimental values was demonstrated for the set of two-junction Josephson interferometers, including partial loops of superconducting sigma and Gauss neurons. The experimental TF of a sigma neuron was successfully fitted on the basis of calculated inductances, which reveals the high potential of the 3D-MLSI software tool for the design of superconducting neurons as well as superconducting electronics devices in general.

## Appendix A

**Figure 13:**
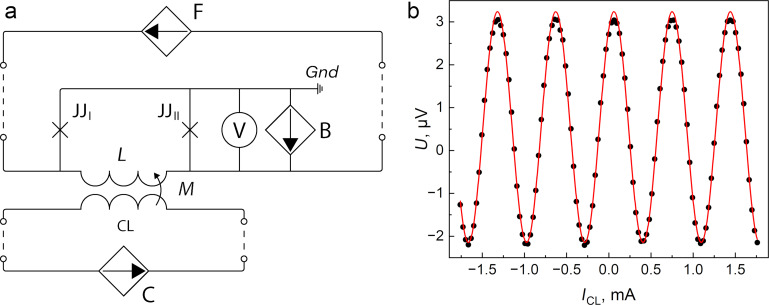
a) A principal scheme of inductance measurements. “B”, “C”, and “F” are current sources. Crosses indicate Josephson junctions JJ_I_ and JJ_II_, which are included in the loop of the two-junction SQUID. *L* stands for the SQUID loop inductance, and *M* is its mutual inductance with the control line CL. b) Typical voltage–flux curve of the investigated SQUIDs. The red curve shows the approximation by a sinusoidal dependence carried out to determine the period.

To measure inductance and coupling of a two-junction SQUID loop, we connect the latter to a current source “B” for biasing and a voltmeter “V” for voltage measurement in a four-point scheme (see Figure 13a). The source “B” supplies a current slightly above the critical one, causing the SQUID to operate in the resistive state. To vary the magnetic flux through the SQUID loop, current sources “C” or “F” are used. When the control current is applied using these sources, the SQUID voltage changes periodically with a period corresponding to one flux quantum. The value of self- or mutual inductance is determined as the ratio of the magnetic flux quantum Φ_0_ to the period of the experimental curve. The type of obtained value depends on the circuit configuration used for the control current source (either “C” or “F”). When using the “C” source, the control current passes through the control line “CL”, which is inductively coupled to the SQUID loop. This way, the mutual inductance *M* between the SQUID loop and the control line “CL” is determined. When using the source “F”, the control current flows via the feedback line connected to the SQUID loop. In this case, the period is determined by the loop inductance *L*. This method is widely used for measuring of self- and mutual inductance of superconducting strip lines [53–54].

It can be noted that the connection of the “B” source to the SQUID is asymmetric in Figure 13a, which can lead to asymmetry in the voltage–flux curve. This does not affect inductance measurements since the period of the voltage–flux curve corresponds to one quantum of applied magnetic flux as determined by a sinusoidal current–phase relationship assumed for tunnel-type junctions. Moreover, the experimental curves were quite symmetrical (see Figure 13b), which allowed for sinusoidal approximation and period determination with an accuracy of the order of 1%. Difficulties could only arise when measuring mutual inductances less than 10 fH, for which it was not possible to record even a single period of the voltage–flux curve due to some limitations related to the large control current (e.g., sample overheating, null voltage drift, and vortex motion). Such weak coupling occurs for distant conductors interacting via the superconducting screen (in particular, the “CL” and “sq” elements in Figure 5). In the case of ultrasmall inductances, measurements were performed using a magnetic flux compensation method with a feedback algorithm [26]. In this method, both sources from the circuit in Figure 13a are used: The “C” source sweeps the control signal, while the “F” current is varied to maintain a constant voltage across the SQUID with an accuracy of 0.1–0.3 μV. A similar method was used for the measurements of TFs of neuron samples [26–27].

## Appendix B

**Figure 14:**
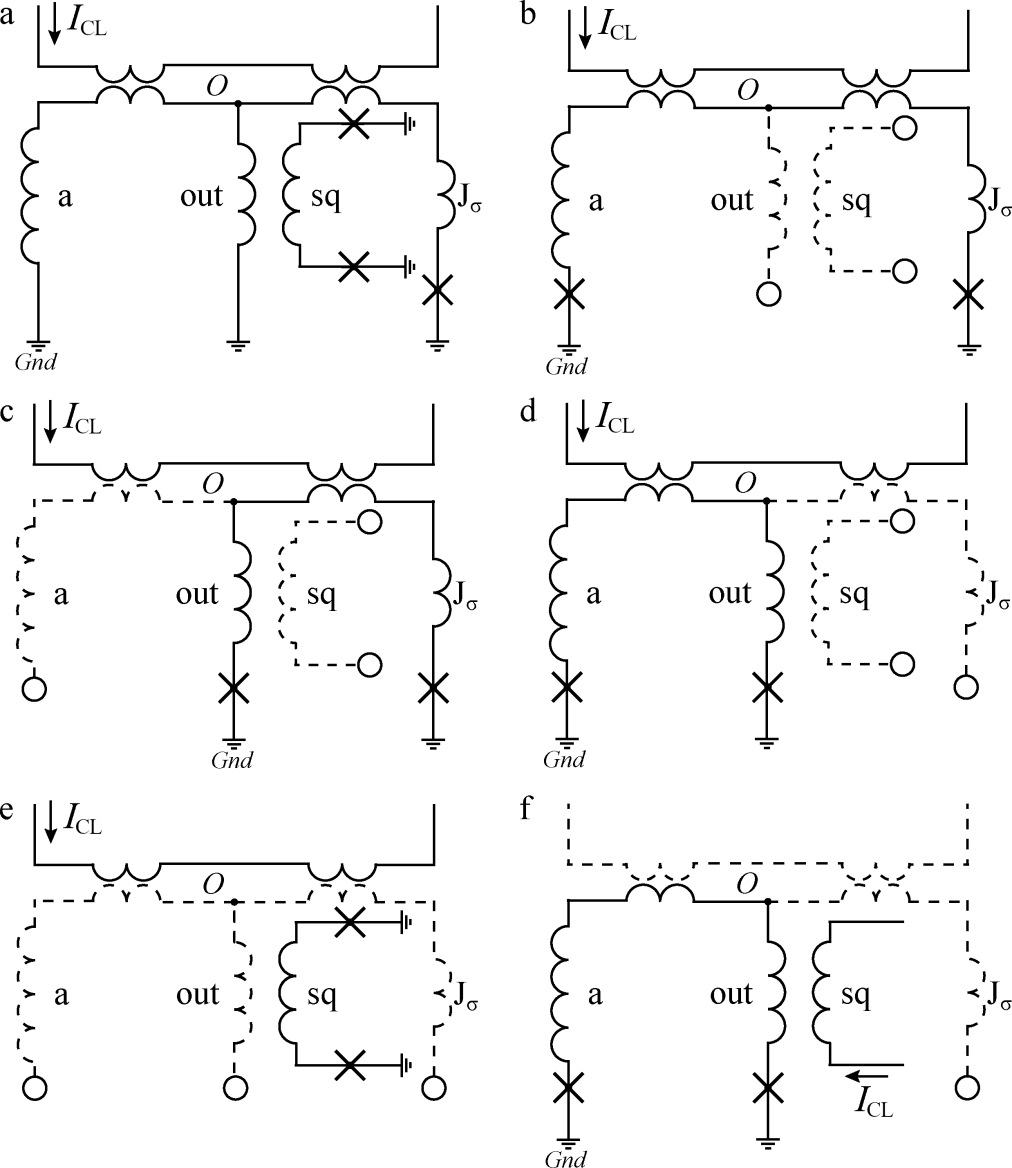
Schematic representation of a sigma neuron and examples of its partial loops (see details in text). JJs are marked with crosses, and the wavy lines stand for inductances. Circles indicate the nodes where the circuit is open, and the corresponding arms are drawn with dashed lines, as well as the elements “CL” and “sq” in case they are not used.

To measure an inductance of the arms of, to be specific, a sigma neuron, the latter should be transformed into a two-junction SQUID coupled to some kind of a control line. Note that the loop of the sigma neuron (consisting of "J_σ_", *L*_"a"_, and *L*_"out"_ arms in Figure 5a and Figure 14a) is, in fact, connected to the ground plane in three points. To transform the neuron into a two-junction SQUID, one of the connections must be opened, while the other two should be closed via Josephson junctions. This can be done in three ways (see Figure 14b–Figure 14d), resulting in the following partial loops of the sigma neuron: (1) the input loop consisting of elements “J_σ_” and “a” (Figure 14b), (2) the Josephson loop consisting of elements “J_σ_” and “out” (Figure 14c), and (3) the inductive loop consisting of elements “out” and “a” (Figure 14d). The fourth interferometer type is a readout SQUID coupled to a neuron in which all three arms are opened (Figure 14e).

A series of specific samples were made, which are two-junction SQUIDS based on partial loops of the sigma neuron. Then, self- and mutual inductances were measured as described in Appendix A. Additionally, the same structures were simulated using the 3D-MLSI software. While measuring the coupling 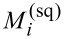 to the readout SQUID loop, the element “sq” (see Figure 5) was used as a control line with its Josephson junctions removed (see Figure 14f for example). As a result, a set of values, *L_k_*, 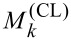, and 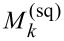 (*k* = 1…4), was obtained. These values are shown in Figure 6. Next, one can express the inductances of the arms as:


[3] $$L=\frac{1}{2}\left(L_{1}+L_{2}-L_{3}\right),$$

[4] $$L_{\text{a}}=\frac{1}{2}\left(L_{1}+L_{3}-L_{2}\right),$$

[5] $$L_{\text{out}}=\frac{1}{2}\left(L_{2}+L_{3}-L_{1}\right).$$

The designations in Equations 3–5 and further correspond to those introduced in [26] to fit the experimental TF. The input mutual inductance,


[6] $$M_{\text{in}}=M_{1}^{\left(\text{CL}\right)}=M_{2}^{\left(\text{CL}\right)}+M_{3}^{\left(\text{CL}\right)},$$

and the output one,


[7] $$M_{\text{out}}=M_{2}^{\left(\text{sq}\right)}=M_{3}^{\left(\text{sq}\right)},$$

were measured directly. Additionally, couplings of each receiving arm (i.e., J_σ_ and a) to the control line were determined as:


[8] $$M_{\text{J}}=M_{2}^{\left(\text{CL}\right)},\;M_{\text{a}}=M_{3}^{\left(\text{CL}\right)}.$$

The screen-mediated coupling of the “CL” and “sq” elements can be defined as *M*_sq_ = 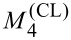. The values defined above allow for a thorough comparison with simulation as well as approximation of the experimental TF shape with a single fit parameter instead of four.

**Figure 15:**
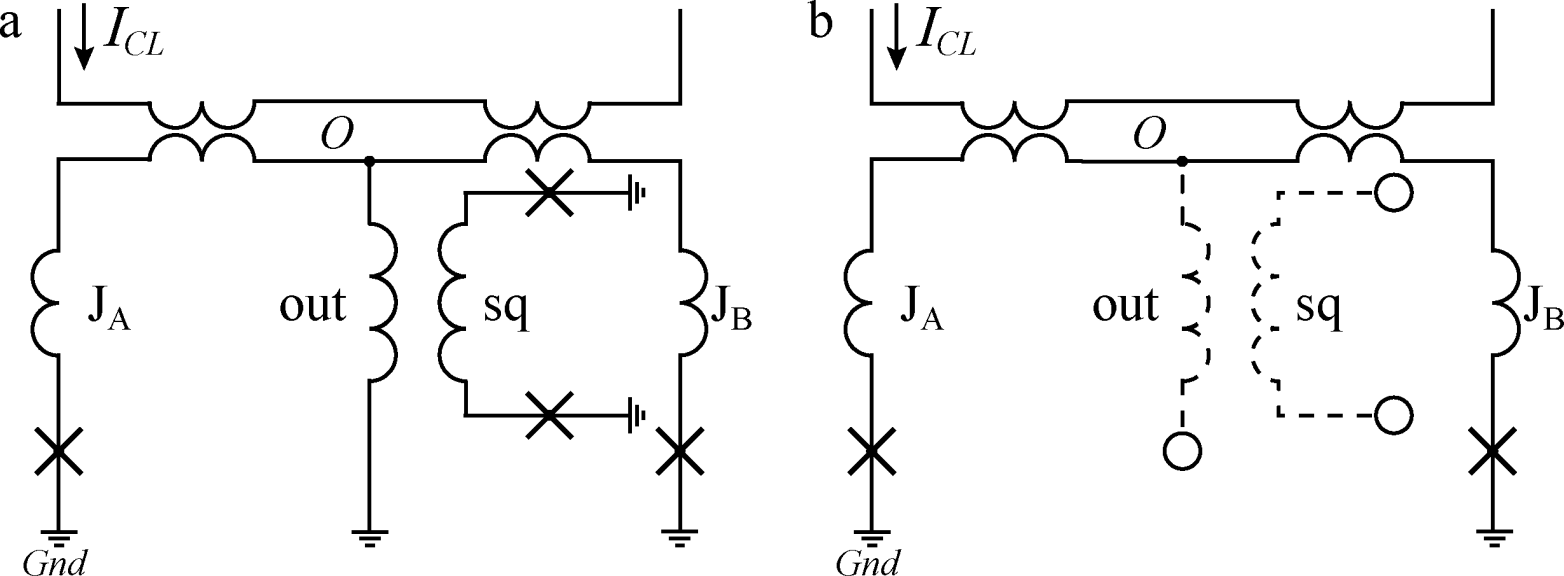
Schematic representation of a Gauss neuron (a) and one of its partial loops (b) (see details in text).

Decomposition of a Gauss neuron (see microphotograph in Figure 5b and the schematic in Figure 15a) yields the same loops upon a change in the designations of elements “J_σ_” and “a” to “J_A_” and “J_B_”, respectively (see Figure 14b and Figure 15b for comparison). For the purposes of Section “Discussion”, the definitions 
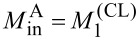
 and 
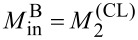
 are to be introduced in accordance with [43], which denote coupling of receiving arms to the control line. The total coupling of the Gauss neuron to the control line is *M*_in_ = 
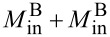
, which was valid within 1% accuracy (also for the sigma neuron).

## Data Availability

Data generated and analyzed during this study is available from the corresponding author upon reasonable request.
